# Augmentation of the Atrophic Mandible with a Block Corticomedullary Graft

**DOI:** 10.1155/2020/6837519

**Published:** 2020-06-28

**Authors:** Layla Louise de Amorim Rocha, Matheus Francisco Barros Rodrigues, Rodrigo da Franca Acioly, Daniel do Carmo Carvalho, Cristofe Coelho Lopes da Rocha

**Affiliations:** ^1^Dentistry Course, Faculdade Cathedral, Boa Vista 69307-053, Brazil; ^2^Department of Oral and Maxillofacial Surgery and Traumatology, Hospital Geral, Boa Vista 69305-455, Brazil; ^3^Department of Infrastructure, Federal Institute, Boa Vista 69303-340, Brazil

## Abstract

The gradual loss of the dental alveolus leads to bone resorption, which may cause atrophy of the maxilla and mandible. One of the most complex procedures in reconstructive surgery is the rehabilitation of patients with atrophic mandibles. Herein, we present a clinical case study of atrophic mandible augmentation with grafts obtained from the iliac crest. The use of reconstruction plates may represent a feasible mechanism for treatment as well as fracture prevention. Mandible augmentation performed by grafting the donor site of the iliac crest showed satisfactory results and resolution of the aesthetic and functional impairments.

## 1. Introduction

Edentulism is a condition that affects a large number of individuals worldwide, especially elderly people [1–3]. The gradual loss of the dental alveolus leads to bone resorption, which may cause atrophy of the maxillae [4]. This atrophy makes the bone fragile and vulnerable to fractures, since the bone mass is reduced [5].

One of the most complex procedures in reconstructive surgeries is the rehabilitation of patients with an atrophic mandible [6]. Factors related to bone quantity and quality, reduced contact area between fractured segments, and inadequate blood supply increase the complexity of this treatment procedure [1]. Several treatment options for these procedures have been reported in the literature, including short implants, bone grafting, and repositioning of the inferior alveolar neurovascular bundle [2].

Techniques for increasing the alveolar dimensions are recommended according to the level of existing bone loss [7]. Monocortical grafts or guided bone grafts are useful for small augmentations, and the interposition graft better suits moderate defects [7]. For defects that are more extensive, osteogenic distraction or reconstruction with grafts from the iliac bone is applied; however, before using these techniques, it is necessary to determine if there is enough bone for distraction [7].

Various types of grafts, including allografts, xenografts, and autografts, are used in the field of dentistry [8]. The autologous bone is considered to be the “gold standard” and the most effective material in bone regeneration procedures owing to its favorable properties and the absence of immunological reactions, showing an optimal combination of osteoconductive, osteoinductive, and osteogenic characteristics when compared to different types of grafts [9, 10]. It may be obtained through donor intraoral areas, such as the maxillary tuberosity, mandibular symphysis, mandibular body, ascending ramus, and zygomatic bone, and through extraoral areas by using the skull bone and the iliac crest [8]. The iliac crest stands out in grafting procedures in cases of atrophy of the jaws due to its high bone volume, relative ease of operation, and low prevalence of morbidity and complications [11]. Graft placement may be performed through intraoral or extraoral access at the discretion of each surgeon [7].

The objective of this study is to present a clinical case involving atrophic mandible augmentation with a graft obtained from the iliac crest, with discussion of the clinical, surgical, and radiographic aspects in addition to a review of the main concepts involved. Considering the possibility of jaw fractures due to severe bone loss, adoption of a grafting procedure can facilitate the patient's functional and aesthetic rehabilitation and fracture prevention.

## 2. Case Presentation

A 72-year-old female patient presented to the dental office with complaints including chewing difficulty and aesthetic dissatisfaction owing to the absence of dental elements. The anamnesis revealed a frustrated rehabilitation attempt approximately 10 years previously, with an overdenture prosthesis in the O-ring on the bottom part under two implants. As time went by, bone resorption occurred.

**Figure 1:**
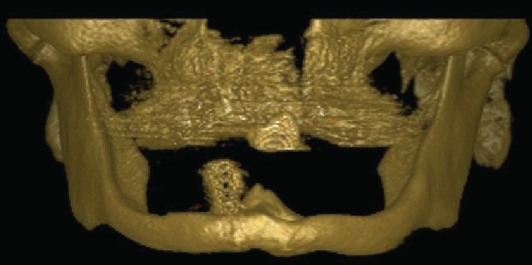
Three-dimensional computed tomography revealing severe mandibular atrophy with perforations at the implant sites.

The patient underwent a computed tomography scan with 3D reconstruction and orthopantomography. Clinical and radiographic examinations revealed severe mandibular atrophy with bilateral exposure of the lower alveolar nerve, perforations at the implant sites (Figure 1), and a bone height of 7 mm (Class VI, Cawood and Howell). In order to obtain assistance during the surgical procedure and to optimize the preoperative planning, a mandibular biomodel was made, which enabled manipulation of the reconstruction plate before the surgical procedure.

Rehabilitative surgical treatment was planned using bone blocks obtained in the iliac crest region to be positioned in the mandible in order to achieve bone height and avoid mandibular fracture [7].

**Figure 2:**
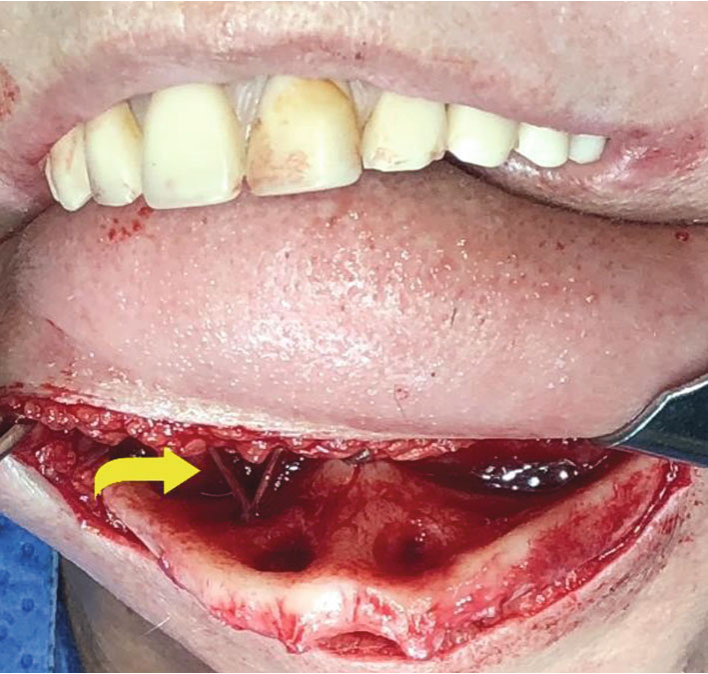
Mandible with severe bone resorption and exposure of the inferior alveolar nerve (yellow arrow).

**Figure 3:**
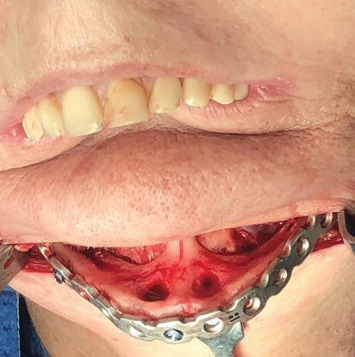
Fixation of the reconstruction plate.

The patient received general anesthesia and was administered corticosteroids (dexamethasone 8 mg intravenously) and anti-inflammatory (tenoxicam 40 mg endovenous) and analgesic (dipyrone sodium 1 g endovenous) drugs after anesthetic induction. For the operation, extraoral access was chosen, with a submental incision followed by divulsion and detachment, exposing the mandible and visualizing the areas of exposure of the alveolar nerve. The nerves were disinserted with the aid of a piezoelectric device, after which the reconstruction plate with 16 holes was fixed using seven fixed screws, three in the region of the genial spines and two on the angles of the mandible intended to prevent fractures and reinforce the mandible to make it functional (Figures 2 and 3). The presence of perforations corresponding to the site of the implants was observed (Figure 2).

**Figure 4:**
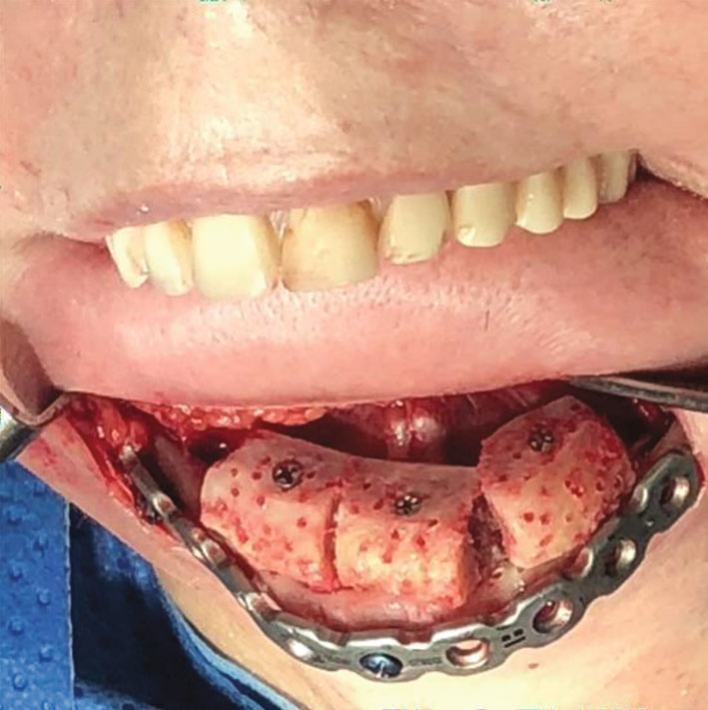
Fixation of the graft.

The graft removed from the iliac crest was decorticalized, and its bed was perforated in order to expose the medullary part to facilitate neovascularization. Then, grafting of the three blocks from the iliac crest was performed with one screw in each block (Figure 4).

**Figure 5:**
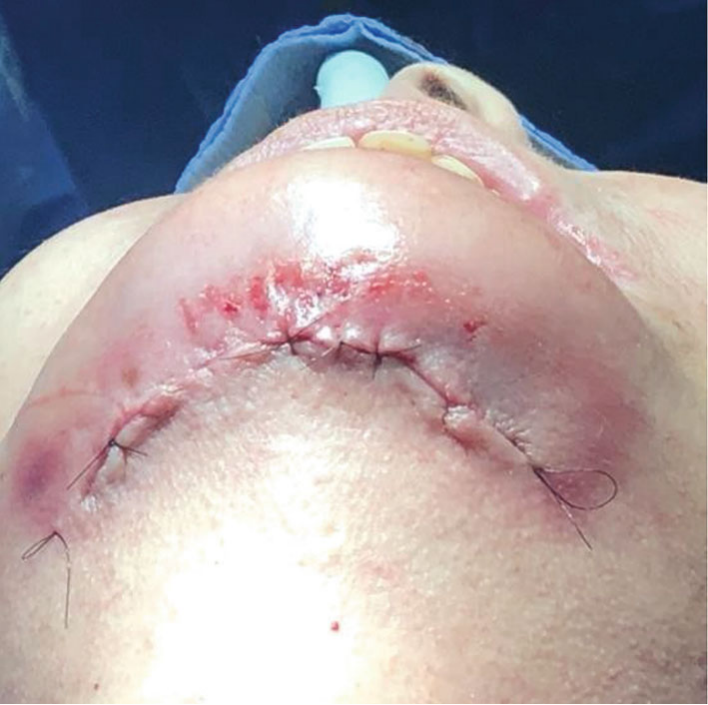
Tissue repositioning with a synthesis operation on the suprahyoid muscles.

For osteoconduction, bioactive NovaBone putty was applied to the bone cement blocks in order to protect the graft and fill the spaces between the bone blocks. Finally, tissue repositioning was performed with a synthesis operation on the suprahyoid muscles by using simple stitches interrupted with Monocryl 4.0 thread and skin and intradermal sutures with 5.0 nylon thread and interrupted simple reinforcement stitches (Figure 5).

**Figure 6:**
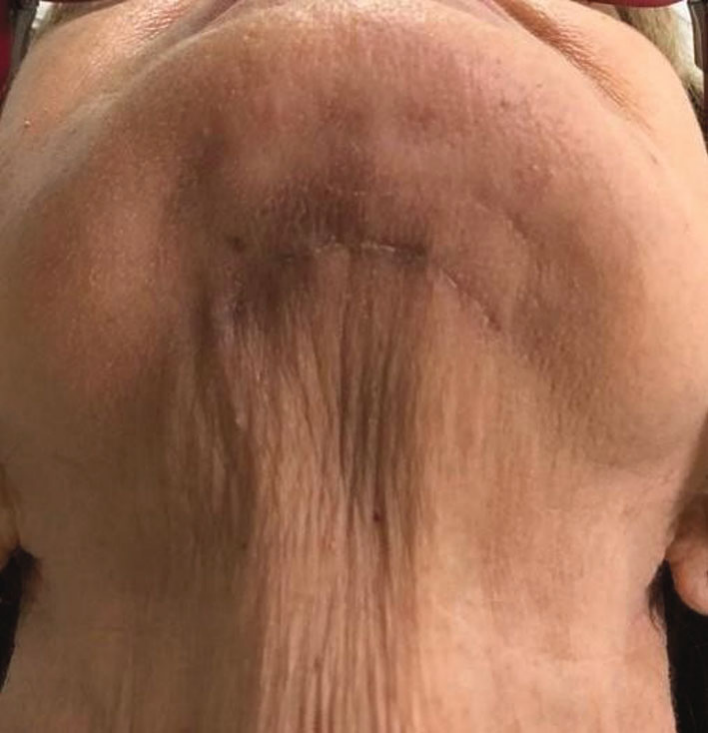
Postoperative healing of the extraoral access site (2 months).

The patient remained hospitalized for 2 days, and the postoperative medication protocol consisted of antibiotics (amoxicillin 500 mg every 8 hours for 7 days), corticosteroids (dexamethasone 4 mg every 12 hours for 3 days), anti-inflammatory (tenoxicam 20 mg every 12 hours for 5 days), and an analgesic (dipyrone sodium 500 mg every 6 hours for 3 days) in case of pain or discomfort. To assist in the treatment of neuralgia, Citoneurin was prescribed. During the postoperative period, the patient reported mild-to-moderate pain and mild paresthesia for the first few days, with no progression of the condition, besides presenting with edema and ecchymosis, which are clinical signs eventually observed in these cases. On the seventh postoperative day, the sutures were removed. After waiting 20 days in order to restore masticatory function, the old prosthesis was repackaged and adapted for the patient to use while waiting for placement of the implants. The extraoral access site showed satisfactory healing and no sequelae (Figure 6).

**Figure 7:**
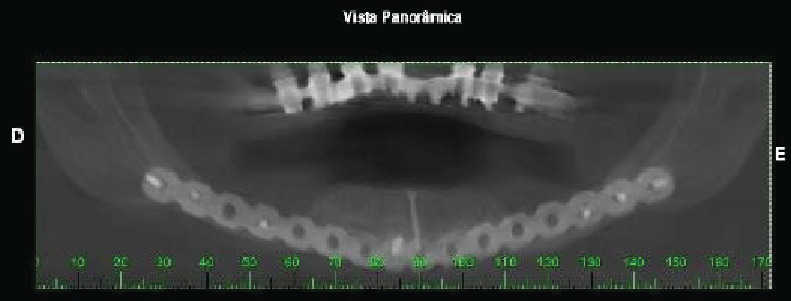
Postoperative panoramic radiography (6 months) demonstrating increased bone height.

**Figure 8:**
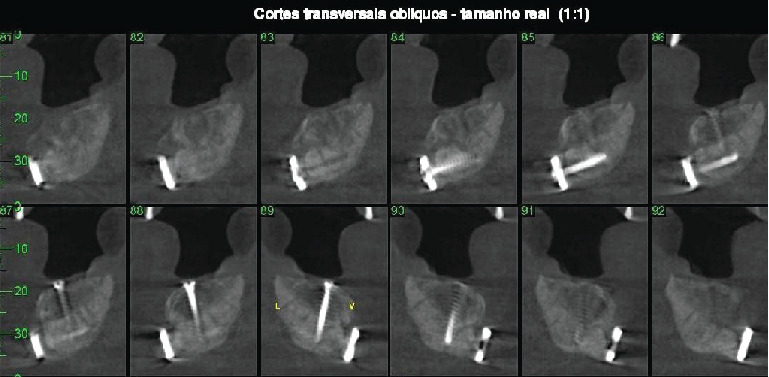
Postoperative computed tomography (6 months).

After four months, the patient returned complaining of pain as a result of the side screws. These were removed to avoid graft exposure and provide comfort. The patient was followed for 6 months, at which point imaging studies revealed successful grafting, and an increase of 9 mm was seen in the jawbone height, which indicated favorable conditions for beginning the dental implant process (Figures 7 and 8).

## 3. Discussion

There are several reports in the literature describing the occurrence of fractures in patients with mandibles showing severe bone resorption [3]. This condition makes the bone fragile and vulnerable to fractures since the bone mass is reduced [5]. Therefore, the surgeon must employ techniques that help prevent fractures in addition to reinforcing the mandible to ensure its functionality.

The therapeutic choice must be based on the patient's age, the severity of the case, and the condition of the bone involved [12]. Although the surgical procedure for insertion of reconstruction plates requires more time, it represents a viable approach both for treatment and fracture prevention [12].

Moreover, the time required for the procedure can be optimized by making a biomodel [13], which, in addition to reducing the incision length, allows visualization and a better understanding of the anatomical region of interest [14] and allows the plate to be molded preoperatively. Thus, during the surgery, the patient's mandible is easily adapted.

Regarding the surgical access point, there is no evidence favoring one over the other [5]. Intraoral access has some disadvantages, such as the presence of the mental nerve on the upper part of the jawbone, which could be injured upon incision and dissection, causing hemilabial hypoesthesia, and possible contamination of the fracture by the oral bacterial fauna, increasing the risk of infection during and after surgery [5]. In turn, extraoral access, despite the possibility of injury to the facial nerve or other anatomic structures, provides the surgeon with a direct view of the fracture, reducing the procedure's complexity compared to intraoral access [5, 15].

Lateralization of the lower alveolar nerve is one of the options for prosthetic-based rehabilitation of patients with moderate-to-severe alveolar reabsorption in the posterior region of the jaw [3]. However, there may be postsurgical consequences resulting from this technique such as neurosensory disorders (neurapraxia, axonotmesis, and neurotmesis) [3]. A high degree of mandibular reabsorption implies a risk of fracture, and this approach is, therefore, not recommended in such cases [3].

## 4. Conclusions

The various risks surrounding mandible atrophy necessitate procedures to prevent further damage and ensure effective treatment depending on the bone loss observed. Jawbone augmentation with an iliac crest graft provided satisfactory results in our patient. Extraoral access allowed for a reduction in case complexity and demonstrated satisfactory scarring without aesthetic problems for the patient. Six months after surgery, an increase of 9 mm was found in the jawbone height, which indicated favorable conditions for beginning the dental implant process.
